# Ecofriendly Synthesis of Magnetic Composites Loaded on Rice Husks for Acid Blue 25 Decontamination: Adsorption Kinetics, Thermodynamics, and Isotherms

**DOI:** 10.3390/molecules28207124

**Published:** 2023-10-17

**Authors:** Fozia Batool, Samia Kanwal, Hafsa Kanwal, Sobia Noreen, Mohamed S. Hodhod, Muhammad Mustaqeem, Gulnaz Sharif, Hafiza Komal Naeem, Javeria Zahid, Abdel-Rhman Z. Gaafar

**Affiliations:** 1 Institute of Chemistry, University of Sargodha, Sargodha 40100, Pakistan; samiasharif75@gmail.com (S.K.); hafsakanwal0343@gmail.com (H.K.); sobia.noreen@uos.edu.pk (S.N.); muhammad.mustaqeem@uos.edu.pk (M.M.); javeriazahid1618@gmail.com (J.Z.); 2 College of Biological Sciences and Medical Engineering, Donghua University, 2999 North Ren Min Road, Shanghai 201620, China; 3 Faculty of Biotechnology, October University for Modern Sciences & Arts, 6th October City 12566, Egypt; mshodhod@msa.edu.eg; 4 Department of Chemistry, Government Graduate College for Women, Mandi Bahauddin 50400, Pakistan; gulnazsharif@gmail.com; 5 Department of Botany, University of Agriculture, Faisalabad 38000, Pakistan; komalnaeem0401@gmail.com; 6 Department of Botany and Microbiology, College of Science, King Saud University, Riyadh P.O. Box 11451, Saudi Arabia

**Keywords:** toxic dye adsorbent, iron oxide, rice husk, Acid Blue 25, Fe_2_O_3_@RH composites

## Abstract

Addressing the growing need for methods for ecofriendly dye removal from aqueous media, this study explores the potential of rice husks coated with iron oxide (Fe_2_O_3_@RH composites) for efficient Acid Blue 25 decontamination. The adsorption potential of Acid Blue 25 is analyzed using raw rice husks and Fe_2_O_3_ nanoparticles in the literature, but their enhanced removal capacity by means of Fe_2_O_3_@RH composites is reported for the first time in this study. Fe_2_O_3_@RH composites were analyzed by using analytical techniques such as TGA, SEM, FTIR, BET, and the point of zero charge (pH_(PZC)_). The Acid Blue 25 adsorption experiment using Fe_2_O_3_@RH composites showed maximum adsorption at an initial concentration of Acid Blue 25 of 80 ppm, a contact time of 50 min, a temperature of 313 K, 0.25 g of Fe_2_O_3_@RH composites, and a pH of 2. The maximum percentage removal of Acid Blue 25 was found to be 91%. Various linear and nonlinear kinetic and isothermal models were used in this study to emphasize the importance and necessity of the adsorption process. Adsorption isotherms such as the Freundlich, Temkin, Langmuir, and Dubinin–Radushkevich (D–R) models were applied. The results showed that all the isotherms were best fitted on the data, except the linear form of the D–R isotherm. Adsorption kinetics such as the intraparticle kinetic model, the Elovich kinetic model, and the pseudo-first-order and pseudo-second-order models were applied. All the kinetic models were found to be best fitted on the data, except the PSO model (types II, III, and IV). Thermodynamic parameters such as ΔG° (KJ/mol), ΔH° (KJ/mol), and ΔS° (J/K*mol) were studied, and the reaction was found to be exothermic in nature with an increase in the entropy of the system, which supported the adsorption phenomenon. The current study contributes to a comprehensive understanding of the adsorption process and its underlying mechanisms through characterization, the optimization of the conditions, and the application of various models. The findings of the present study suggest practical applications of this method in wastewater treatment and environmental remediation.

## 1. Introduction

Nowadays, pollution is a major challenge and it is causing many problems for human health. In recent years, there has been a significant increase in global awareness of the environmental consequences of industrial pollution [1]. Due to globalization and urbanization, the standard of living has increased, but they have also taken human life to a dangerous edge [2]. The discharge of industrial waste from factories and industries is full of harmful and toxic chemicals, including heavy metals and, particularly, dyes that give water an unpleasant color and smell [3]. All these pollutants make water unfit for drinking and household use. This makes human survival difficult, as water is the most crucial element for life [4].

A major factor that contributes to water pollution is the textile industry, particularly in Pakistan, where the textile industry is one of the major contributors to the economy of the country. These dyes are mostly released into water bodies and land without treatment. Different sorts of dyes have different physical and chemical properties that affect water in different ways [5,6]. Dyes contain specific chemical structures that influence water chemically and physically [7]. The presence of different dyes also covers the upper surface of water, which makes the penetration of sunlight into the water quite difficult, and, as a result, the photosynthetic activity in the water decreases, which negatively impacts the aquatic ecosystem [8].

Aside from their toxicity, dyes have carcinogenic, mutagenic, and teratogenic effects on living organisms [9]. With the passage of time, they damage the genetic material of living organisms [10]. Azo dyes that contain amine groups are discharged into water bodies and they undergo chemical reduction and produce amine, which is reported to be carcinogenic in nature [11,12].

One of the more noticeable anthraquinonic dyes is Acid Blue 25, which causes skin and eye irritation and may also create respiratory problems. Thus, the decontamination of dyes is of major concern to environmental chemists [13]. Over the years, numerous methods have been developed for the removal of dyes, including precipitation, an evaporative method, electrolytic extraction, reverse osmosis, ion exchange, membrane process filtration, and chemical treatment. These methodologies look attractive at first sight for the removal of environmental pollutants, but in reality are incompatible for this purpose. These methods have a high operative cost, require skilled workers for their operation, and require a huge amount of chemicals during processing, which make these methods incompatible for this purpose. Furthermore, these methods produce huge amounts of sludge during processing, so another problem arises with respect to how to handle this sludge, which is waste in itself [14].

Adsorption is one of the most promising methods because it is easy to operate, ecofriendly, and cost-effective [15]. In this regard, scientists have used a variety of adsorbents to remove these dyes from aqueous media [16]. The adsorption method has gained the attention of researchers for the removal of organic and inorganic pollutants from aqueous media over the last couple of decades. Materials with easy availability and a low cost are preferred for this purpose. In this context, agrowastes are considered to be an important source of adsorption. The binding capacity of these materials can be intensified via physical and chemical treatments and heat therapy.

Rice husks are one of the major agrowaste products, containing 35% cellulose, 25% hemicelluloses, 20% lignin, and 17% ash by weight [17]. They are cost-effective, ecofriendly, and easily available adsorbents that are used for the removal of pollutants [18]. Modified rice husks were used as adsorbents for the removal of crystal violet dye [19].

The use of nanoparticles is also gaining interest in the field of adsorption and environmental protection. Nanoparticles possess a high surface area and bulk properties, which make them a suitable choice for the adsorption of dyes and metals. They easily undergo modification with various chemical groups, which increases their affinity toward target components. As compared to other sorbents, like clay minerals, chelating minerals, and natural zeolites, nanoparticles serve as excellent adsorbents because of their increased selectivity, affinity, and capacity. In addition to all these properties, the usage of nanoparticles for dye adsorption is cost-effective and an easy method for waste disposal with unique size and shape. Iron oxide (Fe_2_O_3_) nanoparticles were also used as an affordable and easily accessible adsorbent for the removal of dyes [20].

Keeping in view the adsorptive potential of nanoparticles and rice husk, an attempt was made to load magnetic Fe_2_O_3_ nanoparticles on the surface of rice husk to synthesize a new sorbent. This novel adsorbent was prepared by using iron oxide and rice husk for the removal of anionic acidic dye (Acid Blue 25). Composites of iron oxide were prepared and coated on the surface of finely ground rice husk to enhance the adsorptive power of these sorbents. Both adsorbents were cost-effective, safe, and easily available, which made this study effective and low-cost. Adsorption parameters such as dye concentration, time, amount of adsorbent, and pH were optimized. Adsorption kinetics and isotherms were applied for the evaluation of data. The maximum percent removal of Acid Blue 25 was found to be 91% with just 0.2 g of the composites. Synthesized composites of Fe_2_O_3_ loaded on rice husk have been shown to boost the potential for decontamination of aqueous media from harmful dyes and can be successfully applied on a large scale to remove these harmful pollutants from the environment.

The goal of the current work was to synthesize an adsorbent with enhanced adsorption potential by modifying raw rice husk through the loading of magnetic composites. Removal of Acid Blue 25 was achieved by employing this new approach with a small quantity of adsorbent, by developing electrostatic interaction between the dye molecule and synthesized adsorbent. Comprehensive characterization and application of various models make it more effective for scientific understanding of the adsorption process and its applicability for environmental challenges related to Acid Blue 25 decontamination.

## 2. Results and Discussion

### 2.1. Characterization of Fe_2_O_3_@RH Composites

#### 2.1.1. FTIR

**Figure 1:**
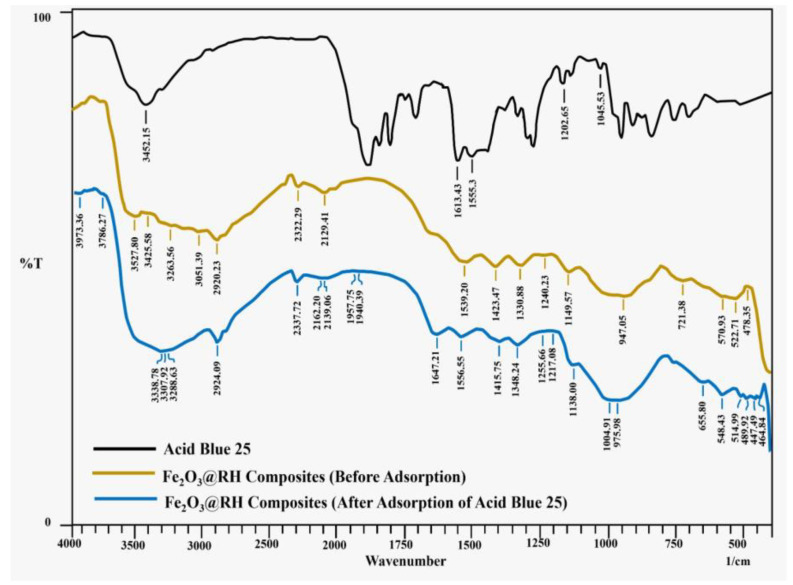
FTIR spectra of Acid Blue 25 and Fe_2_O_3_@RH composites (before and after adsorption).

FTIR spectra of Acid Blue 25 and Fe_2_O_3_@RH composites (before and after adsorption) are shown in Figure 1. Acid Blue 25 gives characteristics peak at 1612 cm^−1^ due to carbonyl stretching of –COOH, and another intense peak at 3452 cm^−1^ appears for –N-H and –O-H stretching of bonds along with intramolecular hydrogen bonding.

The curve before adsorption in Fe_2_O_3_@RH composites shows different peaks than after adsorption. A prominent range of peaks such as 3527, 3425, 3263, and 3051 cm^−1^ indicate the presence of the hydroxyl group. The peak at 2920 indicates the presence of -CH_2_ stretching vibrations. The peaks at 1539, 1423, 1330, and 1149 cm^−1^ indicate the presence of COO^−^ asymmetric stretching, COO^−^ symmetric stretching, C–H bending, and C–O–C stretching of glycosidic linkage, respectively. The range of peaks such as 570, 522, and 478 cm^−1^ indicate the presence of Fe–O bond in Iron (III) oxide.

The curve after adsorption shows various peaks. The peaks in the range of 3500–3200 indicate the presence of O–H and N–H stretching vibrations. The slight shift in this region indicates the adsorption of dye. Moreover, the peak at 2924 indicates methylene stretching vibrations. The peaks at 1556, 1415, 1348, and 1138 indicate the presence of COO^−^ asymmetric stretching, COO^−^ symmetric stretching, C–H bending, and C–O–C stretching of glycosidic linkage, respectively. The appearance of a new peak at 1004 indicates the presence of symmetric stretching of the SO_3_H group present in the dye.

#### 2.1.2. SEM

**Figure 2:**
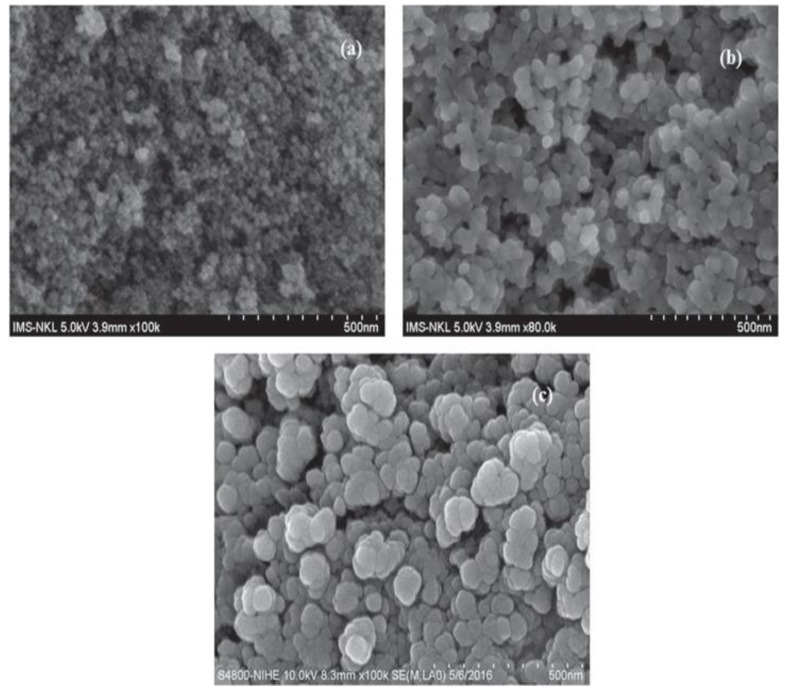
SEM of Fe_2_O_3_@RH composites at 500 nm resolution (taken at three different angles, (**a**) (3.9 mm × 100), (**b**) (3.9 mm × 80 and (**c**) (8.3 mm × 100).

SEM images of the composites are shown in Figure 2, with the large surface area which is responsible for the adsorption of dye on it. It shows the circular or oval shape of the composites. Some pores are also present on it. The presence of a functional group on the composite is responsible for the adsorption of dye on it. The large surface area and presence of cavities on the surface of the adsorbent make it an appropriate surface to attach Acid Blue 25.

#### 2.1.3. pH(PZC)

**Figure 3:**
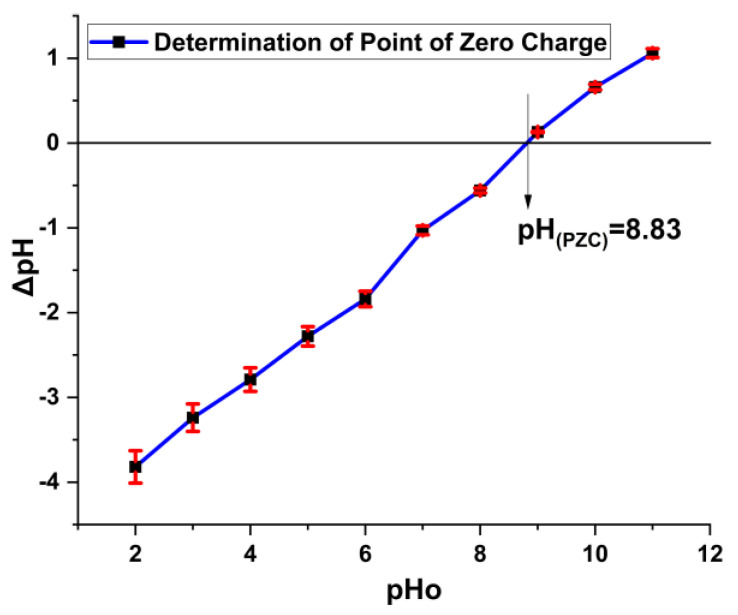
pH(PZC) determination of Fe_2_O_3_@RH composites (before adsorption).

Point of zero charge bears an important place in adsorption study as pH is one of the governing factors for the adsorption of dye on the surface of the composites. During adsorption, electrostatic forces play an important role for adsorbing dye along with other interactive methods. In instances where the pH of an aqueous solution falls below the point of zero charge (PZC) [pH < pH(PZC)], the surface of the Fe_2_O_3_@RH composites adopts a positive charge. In scenarios where the pH of the aqueous solution corresponds to the point of zero charge (PZC) [pH = pH(PZC)], the surface of the Fe_2_O_3_@RH composites maintains an electrically neutral state. Conversely, when the pH of the aqueous solution surpasses the point of zero charge (PZC) [pH > pH(PZC)], the surface of the Fe_2_O_3_@RH composites attains a negative charge. Acid Blue 25 bears negative charge in solution, so it needs a positive surface for attachment. In this regard, dye adsorption is favorable at low pH value where composites carry positive charge on the surface (Figure 3).

#### 2.1.4. Brunauer–Emmett–Teller (BET)

The Brunauer–Emmett–Teller (BET) analysis elucidates the surface area of the Fe_2_O_3_@RH composites, revealing a surface measurement of 107.18 m²/g. In this regard, different methods are available for the determination of adsorbent area and porosity for the adsorption purpose. An empirical method used in this regard is the HK method, which requires the gas pressure and volume of the gas adsorbed to measure the surface of the adsorbent. Results of HK methods show a significant pore diameter of 0.432 nm available for the attachment of Acid Blue 25 on the surface of the adsorbent (Table 1).

Similarly, the SF method, which is considered suitable for solid materials, was also used to determine the surface properties of the adsorbent. Parameters calculated by this method were also found to be in good agreement with the HK method. The porosity of the adsorbent as determined by these two methods is an indicator of small surface pores available on the surface of the Fe_2_O_3_@RH composites.

**Figure 4:**
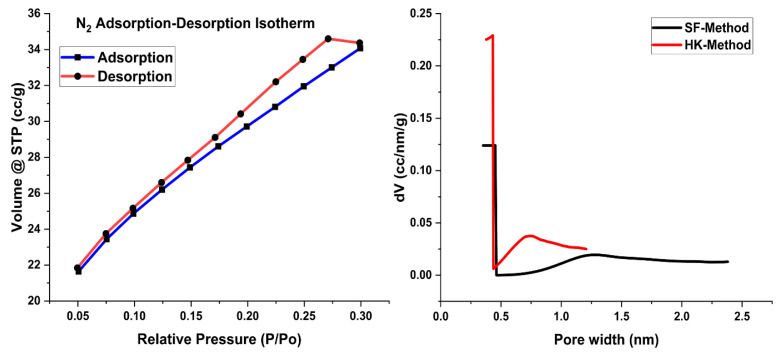
BET plots with nitrogen adsorption–desorption isotherms and pore size distribution curves.

The N2 adsorption–desorption isotherm exhibited characteristics consistent with Type IV, featuring a hysteresis loop labeled as H3, as given in Figure 4. This observation serves as an indicative marker of the coexistence of iron oxide with rice husk within the composite material. Pore size and pore volume, as indicated in Table 1, make the adsorbent favorable for the adsorption of dye.

#### 2.1.5. Thermogravimetric Analysis (TGA)

**Figure 5:**
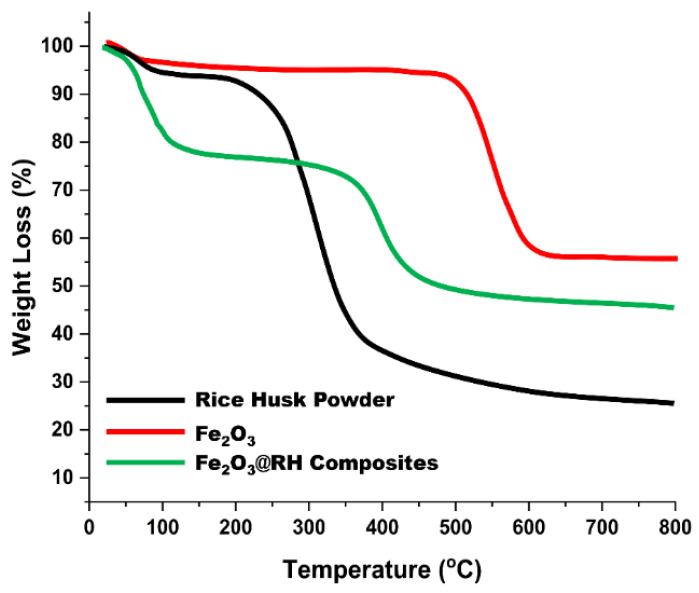
TGA of raw rice husk, Fe_2_O_3_, and Fe_2_O_3_@RH composites.

Thermogravimetric analysis of the raw rice husk, Fe_2_O_3_ nanoparticles, and Fe_2_O_3_@RH composites was performed to observe the behavior of these compounds towards variable heating rates. As shown in Figure 5, raw rice husk shows gradual weight loss from room temperature to 150 °C due to the moisture content and volatile content present in it. Major weight loss occurs at 210 °C, which can be attributed to major pyrolysis of rice husk, which results in main weight loss. For Fe_2_O_3_ nanoparticles, a slight change in weight is observed initially due to moisture content. Major changes in weight occur at 505 °C due to phase transition in synthetic nanomaterials. Fe_2_O_3_ changes to FeO above 500 °C in the second step of the thermogravimetric curve. In the case of Fe_2_O_3_@RH composites, the TGA curve shows weight loss due to moisture contents present in it at 106 °C. Major weight loss occurs at 360 °C due to decomposition of the composites. Due to the loading of Fe_2_O_3_ nanoparticles, the pyrolysis path of the rice husk changes and shifts to high temperature. At this stage, composites break down into their components, and pyrolysis of rice husk takes place [21,22].

### 2.2. Adsorption Parameters

**Figure 6:**
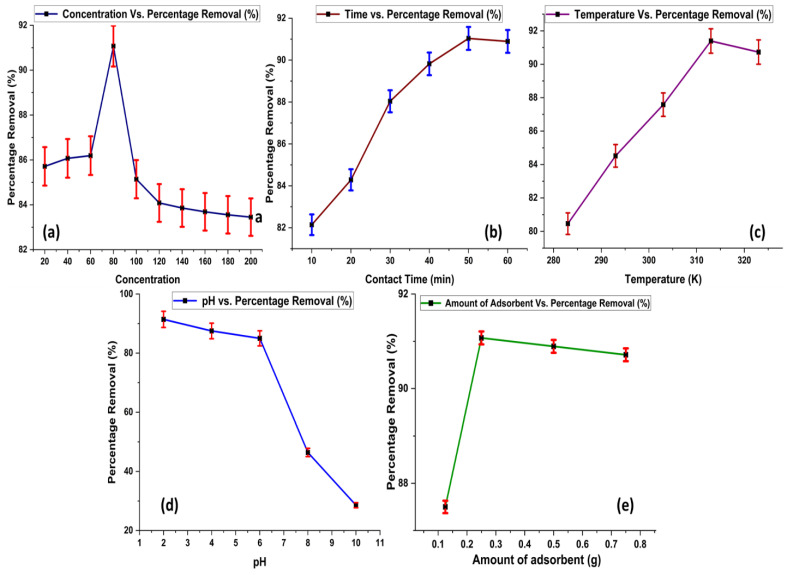
Effect of adsorption parameters on % adsorption of Acid Blue 25, (**a**) Initial Concentration (**b**) Contact Time (**c**) Temperature (**d**) pH and (**e**) Amount of Adsorbent.

An adsorption experiment was performed at variable initial concentration, contact time, temperature, pH, and amount of adsorbent, as shown in Figure 6.

#### 2.2.1. Effect of Initial Concentration of Acid Blue 25

The effective removal of Acid Blue 25 increases as the dye concentration rises. The highest removal rate was observed at a dye concentration of 80 ppm, as shown in Figure 6a. However, beyond this point, increasing the dye concentration does not lead to further increase in adsorption. This suggests that the adsorbent becomes saturated at a dye concentration of 80 ppm. Further increase in concentration of dye have no major effect on adsorption rate. A significant number of vacant positions is available on the surface of the adsorbent, but as dye concentration increases, these positions are captured by molecules of dye. Thus, at concentrations higher than 80 ppm, no promising effect is observed in term of dye adsorption. A similar trend is also observed in the reported data for the removal of Acid Blue 25 [23].

#### 2.2.2. Effect of Contact Time

The adsorption of Acid Blue 25 becomes more pronounced as the duration of contact time between the adsorbent and the dye increases (Figure 6b). The optimal removal of the dye is observed at a contact time of 50 min. However, extending the contact time beyond this duration does not result in any further increase in dye adsorption. This indicates that the maximum adsorption efficiency is achieved at 50 min of contact time. As equilibrium is achieved at this time interval, further prolongation of time gives no profit in terms of adsorption.

#### 2.2.3. Effect of Temperature

Temperature also plays an important role in adsorption studies. As the temperature rises, the percentage removal of Acid Blue 25 also increases. The optimum removal of the dye is achieved at 313 K. As temperature increases, entropy of the adsorption system also increases, which leads to a rise in the adsorption rate. However, raising the temperature beyond this point no longer results in further enhancement of dye adsorption, as depicted in Figure 6c.

#### 2.2.4. Effect of pH

The highest removal of Acid Blue 25 occurs at a pH of 2, as obtained from experimental results given in Figure 6d. This indicates that the adsorbent’s surface and the dye molecules interact most effectively when the solution is acidic. As the pH increases, the surface charges of both the adsorbent and the dye molecules change. As supported by zeta potential, adsorbent surfaces bear positive charge below PZC value, so negatively charged dye molecules give effective removal at pH values below the PZC by develops attractive forces with dye. However, when pH is increased, a decrease in adsorption occurs due to a change in charge on the surface of the adsorbent, which leads to weaker interactions, causing a decrease in the removal efficiency of the dye. Data available in the literature also support the experimental findings of the present study [24].

#### 2.2.5. Effect of Amount of Adsorbent

By increasing the amount of adsorbent, the removal efficiency of the dye increases, but the maximum efficiency is attained at 0.25 g. Further increasing the amount of the adsorbent does not increase the removal efficiency of the dye, as the maximum amount of the dye is removed at 0.25 g, and increasing the amount will not have any effect on the removal efficiency. This major change in adsorption occurs as dye molecules obtain more surface to attach with active sites. However, once a significant amount of dye removal is achieved, then the amount of adsorbent will not have a promising effect on the process of adsorption, as revealed in Figure 6e.

### 2.3. Thermodynamics

Experimental data were collected at various temperatures (ranging from 283 K to 323 K) to assess the influence of temperature on the adsorption process. The thermodynamic property can be described using the subsequent equation:

$$\ln K_{d}=\frac{\Delta S{}^{\circ}}{R}-\frac{\Delta H{}^{\circ}}{\mathrm{RT}}$$

The symbols used in the equation represent the following: ΔS° stands for the alteration in entropy, ΔH° signifies the alteration in enthalpy, R denotes the universal gas constant, with a value of 8.314 Jmol^−1^K^−1^, T represents the absolute temperature, measured in kelvin (K), and K_d_ denotes the distribution coefficient, which is expressed through the subsequent equation:

$$K_{d}=\frac{q_{e}}{C_{e}}$$

Furthermore, the alteration in Gibbs free energy (ΔG°) can be determined through the application of the subsequent equation:

$$\ln K_{d}=\frac{-∆G{}^{\circ}}{\mathrm{RT}}$$

ΔG°, ΔH°, and ΔS° are computed based on the intercept and slope (Figure S1) obtained from the plot correlating to 1/T and lnK_d_ [23]. Equations used for the calculation of different thermodynamic parameters are given in Table S1.

Thermodynamic parameters calculated for Acid Blue 25 are given in Table 2. Results indicate a negative value of Gibbs free energy (ΔG°) with increase in temperature. A negative value of Gibbs free energy is due to spontaneous nature of adsorption reaction. The value of Gibbs free energy increases as temperature is decreased; this reveals that adsorption is more favorable at high temperature ranges. A similarly positive outcome of enthalpy and entropy for the reaction is an indicator of an exothermic reaction nature and an increase in randomness during the adsorption process, respectively. As dye molecules attach to the surface of the adsorbent, there is an increase in randomness at the solid/solution interface which leads to an increase in entropy (ΔS°), and similar behavior has been observed by other researchers for dye adsorption. As the temperature is increased during adsorption, the movement of molecules also increases. This leads to the attachment of dye molecules on the surface of active sites [25]. Furthermore, it is also reported in the literature that the temperature has a positive effect on the adsorption process by increasing the area of the adsorbent. Pore volume increases and the probability of dye molecules to attach with the surface of the adsorbent also increases [26].

### 2.4. Adsorption Isotherms

Equilibrium data of adsorption are used to check the design and operation of sorption processes. They also predict the performance of the sorption contact processes under a range of operating conditions. Large numbers of adsorption isotherms are available in this regard; however, few are found to be quite suitable for the purpose, i.e., Langmuir and Freundlich. In the present study, we applied Langmuir, Freundlich, and Dubinin–Radushkevich isotherms to our work. The adsorption isotherms applied to our work are given in Table S2.

Results indicate a high R^2^ value of 0.9 for all adsorption isotherms except the linear form of D–R isotherm, which gives 0.8 R^2^ for the adsorption of Acid Blue 25, as indicated in Table 3.

**Figure 7:**
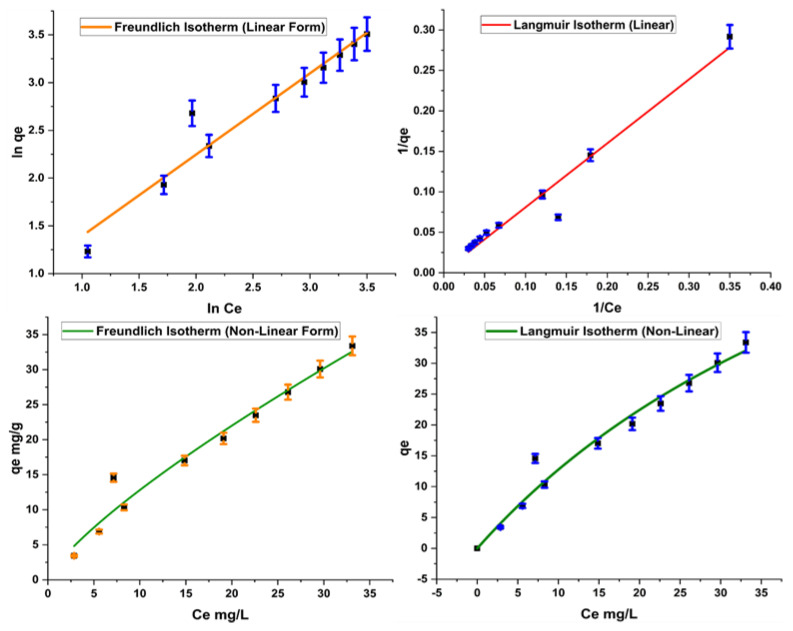
Linear and nonlinear forms of Freundlich and Langmuir isotherms.

The Freundlich isotherm assumes multilayer adsorption mechanism for the adsorption. K_F_ and 1/n are Freundlich constants showing adsorption capacity and adsorption intensity, respectively. Adsorption is considered favorable if the value of K_F_ is between 1 and 20. Similarly, a low value of 1/n is an indicator of a heterogeneous surface suitable for adsorption purpose. Results obtained for the present study show efficient adsorption based on Freundlich constants (Figure 7). Values of K_F_ and 1/n also depict a favorable adsorption of Acid Blue 25 on the adsorbent.

On the other hand, Langmuir is based on the assumption of the unilayer adsorption phenomenon. The constants in Langmuir, q_m_ and R_L_, give maximum monolayer adsorption capacity and Langmuir affinity parameter, respectively. A high value of q_m_ represents maximum adsorption, and adsorption is considered favorable if R_L_ value is low. Results of the Langmuir isotherm, as given in Table 3, indicate that the reaction is promising for adsorption.

As far as the D–R model is concerned, it is used to determine the nature of the adsorption reaction. The D–R model provides information on whether the adsorption reaction is physical, chemical, or ion exchange in nature. The value of E is calculated for this model; an E value less than 8 kJ/mol indicates the physical nature of adsorption, and E = 8 to 16 kJ/mol is an indicator of the ion exchange mechanism. If the E value is found to be higher than 16 kJ/mol, then the adsorption process is chemisorption in nature. The current study gives a significantly high value of E, which is a sign of the chemical nature of the adsorption of dye on the surface of the adsorbent.

**Figure 8:**
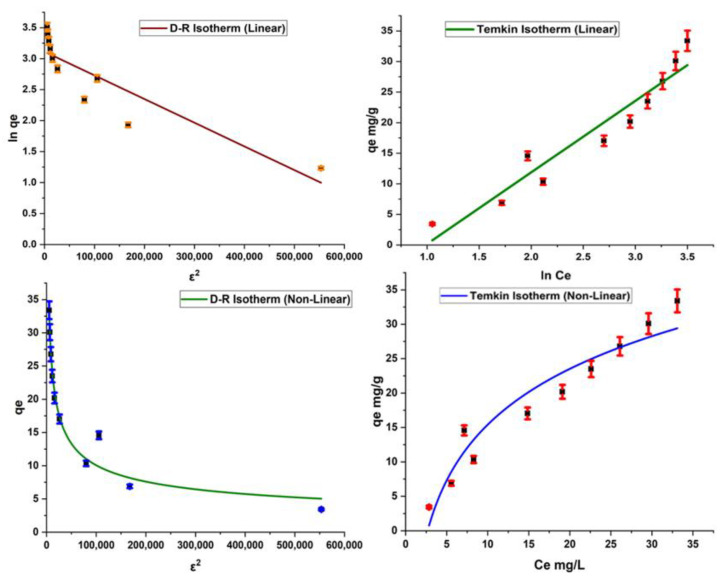
Linear and nonlinear forms of Dubnin and Temkin isotherms.

The Temkin isotherm is based on the assumption that the heat of the reaction changes during the adsorption phenomenon (Figure 8). The Temkin constant K_T_ contributes information about binding energy in the adsorption process, and the constant B_T_ provides information about the heat of adsorption [27]. Results indicate that B_T_ > 0, which expresses the exothermic nature of the adsorption process, as given in Table 3.

### 2.5. Adsorption Kinetics

The time required for a particular adsorption process and the uptake level of the adsorption phenomenon are described by adsorption kinetics. Several kinetic models are available in this regard, as given in Table S3.

**Figure 9:**
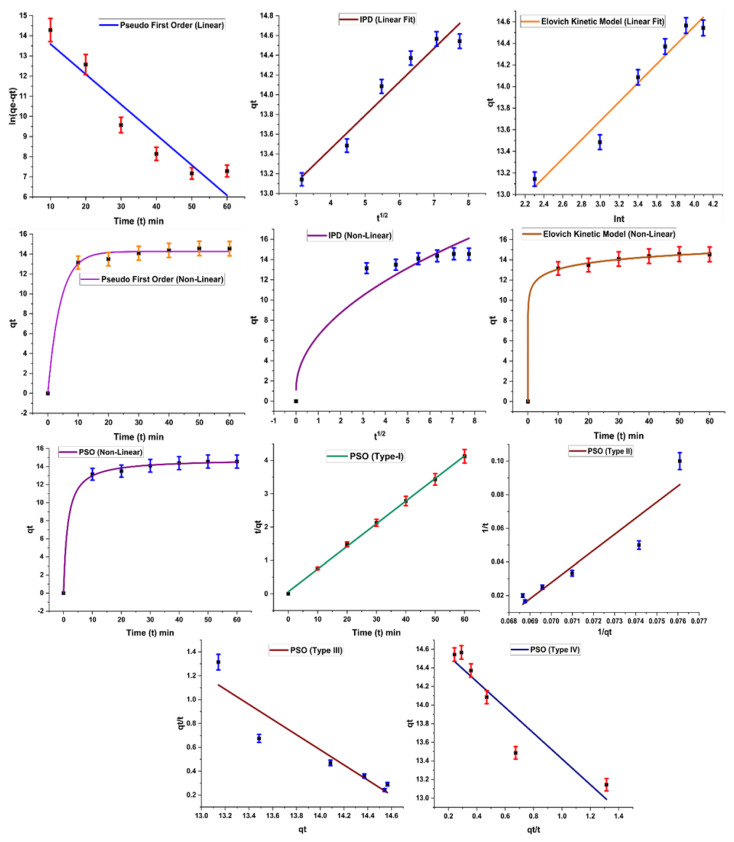
Kinetic studies applied on the adsorption of Acid Blue 2.

Figure 9 indicates different forms of kinetic models based on the abovementioned equations. R^2^ value was calculated for each kinetic model, and a value close to 1 is an indicator of model applicability for the adsorption system.

Results of kinetic models are summarized in Table 4, which indicates the successful application of the pseudo-first-order kinetic model on the experimental results, with a high R^2^ value (0.9 for linear and 0.99 for nonlinear form). According to the pseudo-first-order kinetic model, rate of adsorption is related to adsorption sites freely available on the surface of the adsorbent. As these sites become occupied, the adsorption rate also falls. A similar trend is observed in the present study: adsorption increases with the increase in contact time initially, but after maximum adsorption, no significant role is played in terms of adsorption rate by increasing time. A similar trend is also reported in the literature by other researchers [25].

In the case of the pseudo-second-order kinetic model, only type I gives a good result, with R^2^ close to 1; other types have low values for R^2^. This model is based on the assumption that the rate-limiting step is the adsorption phenomenon, which is responsible for attractive forces developing between the adsorbent and dye molecules [28]. The Elovich kinetic model is based on the theory that the rate of adsorption of dye decreases exponentially as the amount of dye adsorbed increases. It also provides information about the nature of the adsorption process. This model was also applied successfully on the work, indicating chemisorption behavior of the adsorbent.

Kinetic data were also subjected to the intraparticle diffusion kinetic model suggested by Weber and Morris. A high value of regression coefficient (R^2^ = 0.94) was obtained for the model, with value of adsorption capacity, C, showing significant adsorption of the dye. However, the plot of the diffusion model shows that the line is not passing through the origin, which is an indicator of the fact that the intraparticle diffusion kinetic model is not the only rate-governing step for the adsorption reaction [29].

### 2.6. Desorption/Regeneration of Fe_2_O_3_@RH Composites

**Figure 10:**
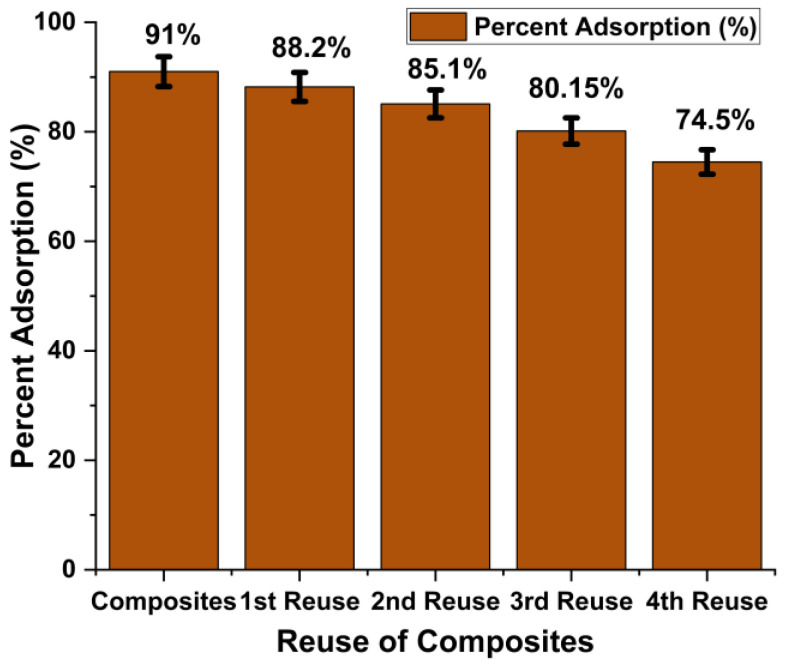
Regeneration of the Fe_2_O_3_@RH composites for dye removal.

Solid waste generated after the adsorption process is another serious threat to the environment. Its proper disposal is quite difficult as it carries harmful dyes on it. In this regard, different efforts are made by scientists to regenerate adsorbent and make it less harmful to the environment [30,31]. In the present work, after the adsorption phenomenon, the adsorbent was regenerated by treating it with ethanol for 1 h. Dye was desorbed, and after filtration and drying, adsorbent was again applied for the removal of dye. Initially, composites showed 91% dye removal efficiency. After four regeneration cycles, adsorption efficiency gradually decreased to 74.5%, as shown in Figure 10. Synthetic composites were found to be quite stable and efficient after several reuses. This property of composites makes them an excellent choice for water decontamination.

### 2.7. Adsorption Mechanism

**Figure 11:**
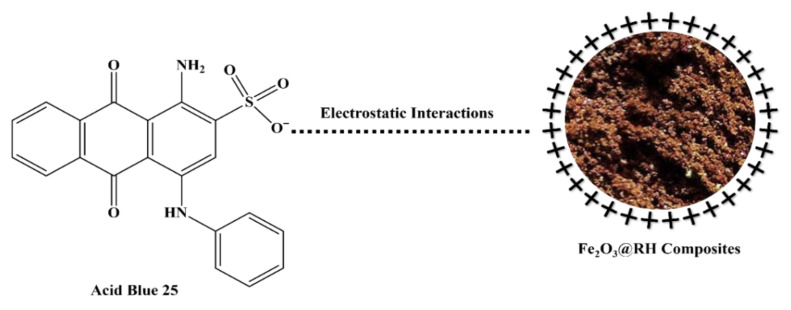
Adsorption mechanism of Fe_2_O_3_@RH composites.

The concept of the point of zero charge elucidates the positive charge present on the surface of the adsorbent, shedding light on the underlying mechanism of adsorption. The determined point of zero charge, calculated to be 8.83, signifies a neutral charge state on the adsorbent’s surface at this pH. However, when the pH falls below this point, the adsorbent’s surface becomes positively charged; conversely, if the pH exceeds the point of zero charge, the surface of the adsorbent takes on a negative charge. As a result, the optimal adsorption of Acid Blue 25 is observed at a pH of 2, indicative of an acidic medium. With an increase in pH beyond this point, the adsorption of Acid Blue 25 diminishes. This phenomenon can be attributed to the reduction in positive charge on the adsorbent’s surface as the pH rises (Figure 11). The adsorption mechanism is largely governed by electrostatic forces of attraction between the dye molecules and the charged adsorbent surface, and this interplay comprehensively elucidates the intricate process of adsorption.

### 2.8. Comparison of Present Study with Reported Data

To evaluate the adsorption potential of synthetic adsorbents for the removal of Acid Blue 25, a comparison was made with reported data, as given in Table 5. Raw rice husk gives 80 to 90% removal of Acid Blue 25 with 2 g of adsorbent added, by increasing time interval up to 2 h. Similarly, Fe_2_O_3_ gives 75.7% dye removal if the amount of adsorbent added is 1 g. In comparison with these adsorbents, currently synthesized Fe_2_O_3_@RH composites give 91% adsorption potential with just 0.2 g of the adsorbent. A small amount of adsorbent providing efficient removal of Acid Blue 25 dye makes it an excellent choice for dye remediation for polluted water. Other data reported for the removal of Acid Blue 25 by employing diatomite show 72.81% removal efficiency with 0.9 g of the adsorbent material utilized.

## 3. Materials and Methods

### 3.1. Materials and Equipment

All chemicals used for the research work were of analytical grade with high purity. A list of chemicals and equipment utilized for the current study is given in Table S4. Rice husk was obtained from the Sargodha Rice Mill, Pakistan.

### 3.2. Methods

#### 3.2.1. Preparation of Rice Husk

The obtained rice husk was washed 4–5 times with Millipore water to completely remove dust and impurities, and then it was dried for two days. After drying, the dried rice husk was crushed and ground into fine powder by using a pestle and mortar and passed through sieves to obtain a finely divided powder of rice husk.

#### 3.2.2. Synthesis of Fe_2_O_3_@RH Composites

**Figure 12:**
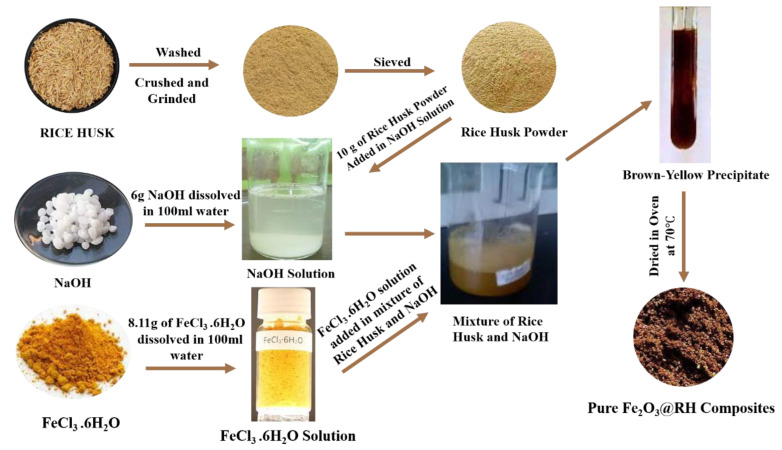
Preparation of Fe_2_O_3_@RH composites.

For the synthesis of Fe_2_O_3_@RH composites, 6 g of NaOH was dissolved in 100 mL of water and added to 10 g of finely divided powder of rice husk in a 250 mL beaker. In another flask, 8.11 g FeCl_3_.6H_2_O was dissolved in 100 mL of water, and the solution was heated to 70 °C to make it hot. The mixture of rice husk and NaOH was heated at 70 °C followed by the slow addition of a hot solution of FeCl_3_.6H_2_O. The slurry was mixed by constant stirring and aerated for 1 h until a brown–yellow color solution appeared. The product was dried in a drying oven at 70 °C for 12 h. The dried product was washed with water to remove excess NaOH, and a pure product was obtained. This final product was dried again for 2 days to remove the moisture. The final product obtained was pure Fe_2_O_3_@RH composites (Figure 12).

$${\mathrm{FeCl}}_{3}\times {6H}_{2}O+3\mathrm{NaOH}\;\to \;{\mathrm{Fe}\left(\mathrm{OH}\right)}_{3}+3\mathrm{NaCl}+{\;6H}_{2}O\;{2\mathrm{Fe}(\mathrm{OH})}_{3}\overset{\mathrm{Heat}\;\mathrm{at}\;70\;{}^{\circ}C}{\to }{\mathrm{Fe}}_{2}O_{3}+{3H}_{2}O$$

### 3.3. Batch Experiment

A 1000 ppm stock solution of Acid Blue 25 dye was formulated by dissolving 1 g of the dye in a 1000 mL round-bottom flask, which was then adjusted to the mark. Following this, a series of Acid Blue 25 solutions with concentrations of 20, 40, 60, 80, 100, 120, 140, 160, 180, and 200 ppm were prepared from the stock solution. These solutions were employed in the subsequent adsorption experiments.

The adsorption investigations for Acid Blue 25 were performed using 100 mL Erlenmeyer flasks, where each flask accommodated 50 mL of the dye solution. The optimization of Acid Blue 25 dye removal was attained by varying adsorption parameters, including pH levels (ranging from 2 to 8), amount of Fe_2_O_3_@RH composite (ranging from 0.125 to 0.75 g), initial concentration of Acid Blue 25 (ranging from 20 to 200 ppm), and contact time (ranging from 20 to 60 min).

During each experimental trial, the solution underwent agitation using an orbital shaker (SHIN SAENG, Model No. SKIR-601L) at a consistent speed of 150 rpm. Subsequently, the residual concentration of Acid Blue 25 was quantified by means of a UV/visible spectrophotometer (Peak Instruments Model No. C-7200, Peak Instruments (Shanghai) Co., Ltd. China) at the wavelength of maximum absorption (λmax) at 600 nm. Each experiment was repeated three times to obtain the mean value. All obtained experimental data were meticulously charted and subjected to analysis using Origin Pro 2022 (Version Number 225 (9.9.0.225)). The assessment of adsorption efficiency (expressed as R (%)) was conducted through the utilization of the subsequent equation [35,36]:

$$R(\%)=\frac{{(C}_{0}-C_{e})}{C_{0}}\times 100$$

The adsorption capacity (q_e_, mg/g) of Acid Blue 25, which signifies the quantity of Acid Blue 25 adsorbed upon reaching equilibrium, was evaluated by using the subsequent equation: [37,38]:

$$q_{e}=\frac{{V(C}_{0}-C_{e})}{m}$$

The variable q_t_ signifies the adsorption capacity of Acid Blue 25 at a specific time (t) and is measured in milligrams per gram (mg/g). The parameter C_0_ corresponds to the concentration of Acid Blue 25 at the initial time (t = 0), measured in milligrams per liter (mg/L). Ce represents the concentration of Acid Blue 25 at the designated time (t) and is expressed in milligrams per liter (mg/L). The symbol V pertains to the volume of the solution in liters (L), and m denotes the mass of the Fe_2_O_3_@RH composite sorbent, quantified in grams (g).

The adsorption data pertaining to Acid Blue 25 dye were subjected to modeling using diverse adsorption isotherms, which encompassed the Freundlich, Langmuir, Temkin, and Dubinin–Radushkevich models. In the kinetic experiments, the initial concentration of Acid Blue 25 was set at 80 ppm, while the experimental volume equaled 50 mL. The Fe_2_O_3_@RH composite mass employed amounted to 25 mg.

Throughout the course of the kinetic experiments, 5 mL of the suspension was extracted at predefined time intervals and subjected to agitation using an orbital shaker. This methodology facilitated the determination of the residual concentration of Acid Blue 25 within the solution. The subsequent equation was employed to compute the amount of Acid Blue 25 adsorbed (q_t_, mg/g) at a specific time (t) [39].

$$q_{t}=\frac{{V(C}_{0}-C_{t})}{m}$$

The experimental dataset underwent analysis through the utilization of four kinetic models: the pseudo-first-order model, the intraparticle diffusion model, the Elovich kinetic model, and the pseudo-second-order model. These models were employed to characterize and comprehend the adsorption kinetics of Acid Blue 25 dye within the scope of the study.

### 3.4. Point of Zero Charge (pH_PZC_)

The point of zero charge (PZC) of the Fe_2_O_3_@RH composites was determined through the utilization of the salt addition method. This method involved establishing a relationship between pH and surface potential over an extensive pH range (from 2 to 11). To execute the experiment, a 0.1 M solution of NaNO_3_ was carefully prepared in a 500 mL flask. Subsequently, 50 mL of this solution was transferred to ten distinct flasks. In each flask, the initial pH values (ranging from 2 to 11) were precisely adjusted using 0.1 M HCl and 0.1 M NaOH solutions. Following this, accurately measured quantities (0.25 g each) of the Fe_2_O_3_@RH composites were introduced into the flasks containing solutions with varying pH levels. These solutions were subjected to thorough agitation on an orbital shaker for approximately 24 h while maintaining a controlled temperature of 30 °C. After the agitation period, the solutions were filtered, and the final pH values were determined using a pH meter. The alteration in pH before and after the experiment was computed using the subsequent equation:

$$∆pH={pH}_{o}-pH$$

In this context, “pH” denotes the final pH value, while “pH_o_” represents the initial pH value. The juncture where these two values coincide on the x-axis marks the point of intersection, which indicates the point of zero charge (PZC) for the Fe_2_O_3_@RH composites [40,41].

## 4. Conclusions

The phenomenon of adsorption emerges as a remarkable solution for effectively removing hazardous substances, including dyes, from aqueous environments. This study highlights the utilization of rice husk coated with iron oxide (Fe_2_O_3_@RH composites) as a highly favorable choice in addressing the removal of toxic Acid Blue 25 dye. Characterization of the composites provides information about important functional groups present on the surface of the adsorbent which help to develop electrostatic interaction between Acid Blue 25 and synthesized composites. Overall results demonstrated that although raw rice husk and Fe_2_O_3_ nanoparticles are separately used for the removal of Acid Blue 25, their removal efficiency was low compared with the current work. A small amount of synthetic composites (0.2 g) was found to be effective for the significant concentration of dye removal (91%) in just 50 min of contact time. Feasibility of the adsorption process and its mode of adsorption was also depicted by adsorption isotherms and thermodynamic models applied successfully to the work. The present work gives an insight into Acid Blue 25 remediation from wastewater with inexpensive adsorbent and promising potential of adsorption.

## Figures and Tables

**Table 1:** BET results with surface area, pore volume, and diameter.

Fe_2_O_3_@RH Composites (Before Adsorption)						
Surface Area (m^2^/g)	SF-Method Micropore Volume (cc/g)	HK-Method Micropore Volume (cc/g)	SF-Method Pore Diameter (nm)	SF-Method Pore Diameter (Å)	HK-Method Pore Diameter (nm)	HK-Method Pore Diameter (Å)
107.18	0.047	0.049	0.4522	4.522	0.432	4.32

**Table 2:** Thermodynamics results for Acid Blue 25.

	Thermodynamics Model		Acid Blue 25 Dye
Parameters	Temp. (°C)	Temp. (K)	Fe_2_O_3_@RH Composites
ΔG° (KJ/mol)	10	283	0.4563
	20	293	−0.2144
	30	303	−0.8667
	40	313	−1.9592
	50	323	−1.8032
ΔH° (KJ/mol)	Calculated from slope		18.330
ΔS° (J/K*mol)	Calculated from intercept		63.353
R^2^	0.92		

**Table 3:** Linear and nonlinear parameters calculated for adsorption isotherms.

Isothermal Models	Acid Blue 25 Dye (Linear Form)	Acid Blue 25 Dye (Nonlinear Form)
Freundlich	Fe_2_O_3_@RH Composites	Fe_2_O_3_@RH Composites
K_F_ (mg/g)	1.719	2.12
1/n	0.851	0.77
R^2^	0.942	0.96
Langmuir	Fe_2_O_3_@RH Composites	Fe_2_O_3_@RH Composites
R_L_	0.913	-
q_m_ (mg/g)	529.10	92.68
K_L_	0.0024	0.0159
R^2^	0.96	0.97
Dubinin–Radushkevich	Fe_2_O_3_@RH Composites	Fe_2_O_3_@RH Composites
q_mDR_ (mg/g)	22.462	1061.33
K_DR_ (mol^2^/kJ^2^)	3.82E-06	−1.40
R^2^	0.8	0.96
E (KJ/mol)	361.79	-
Temkin	Fe_2_O_3_@RH Composites	Fe_2_O_3_@RH Composites
K_T_	0.37	0.3738
R^2^	0.92	0.921
B_T_ (J/mol)	11.69	RT/B_T_ = 11.691

**Table 4:** Kinetics results applied on the adsorption of Acid Blue 25.

Kinetic Models	Acid Blue 25 Dye (Linear Form)	Acid Blue 25 Dye (Nonlinear Form)
Pseudo-first-order	Fe_2_O_3_@RH Composites	Fe_2_O_3_@RH Composites
K_1_ (g/mg.min)	−0.003	0.24
q_e1_ (mg/g)	3.30E6	14.25
R^2^	0.9	0.99
Pseudo-second-order	Fe_2_O_3_@RH Composites (Type I)	Fe_2_O_3_@RH Composites
K_2_ (g/mg.min)	0.072	0.0473
q_e2_ (mg/g)	14.72	14.82
R^2^	0.999	0.99
Pseudo-second-order	Fe_2_O_3_@RH Composites (Type II)	Fe_2_O_3_@RH Composites
K_2_ (g/mg.min)	0.043157516	-
q_e2_ (mg/g)	−1.555209953	-
R^2^	0.88	-
Pseudo-second-order	Fe_2_O_3_@RH Composites (Type III)	Fe_2_O_3_@RH Composites
K_2_ (g/mg.min)	0.1667	-
q_e2_ (mg/g)	9.458	-
R^2^	0.87	-
Pseudo-second-order	Fe_2_O_3_@RH Composites (Type IV)	Fe_2_O_3_@RH Composites
K_2_ (g/mg.min)	0.128	-
q_e2_ (mg/g)	10.72	-
R^2^	0.87	-
Elovich	Fe_2_O_3_@RH Composites	Fe_2_O_3_@RH Composites
α	268539.7755	266024.169
β	1.142857143	1.141
R^2^	0.96	0.99
Intraparticle Diffusion	Fe_2_O_3_@RH Composites	Fe_2_O_3_@RH Composites
C	12.09	1.11
K_diff_	0.33	5.38
R^2^	0.94	0.93

**Table 5:** Comparison of Fe_2_O_3_@RH composites adsorption with reported data.

Adsorbent Used	Amount of Adsorbent	Dye	Adsorption	Reference
Rice Husk	2 g	Acid Blue 25	80–90%	[32]
Fe_2_O_3_	1 g	Acid Blue 25	75.7%	[33]
Fe_2_O_3_@RH	0.2 g	Acid Blue 25	91%	Present Study
Diatomite	0.9 g	Acid Blue 25	72.81%	[34]

## Data Availability

Supplementary data is provided.

## Supplementary Materials

The following supporting information can be downloaded at: https://www.mdpi.com/article/10.3390/molecules28207124/s1. Table S1. Equations used for Calculation of Thermodynamic Parameters; Table S2. Isothermal Models and Equations Applied on Adsorption of Acid Blue 25; Table S3. Kinetic Models Applied on Adsorption of Acid Blue 25; Table S4. Chemicals and Equipment used for the Work; Figure S1. Thermodynamics for Acid Blue 25. Refs. [42,43,44,45,46,47,48,49,50] are cited in the Supplementary Materials.
