# Supplementary material for: Ecofriendly Synthesis of Magnetic Composites Loaded on Rice Husks for Acid Blue 25 Decontamination: Adsorption Kinetics, Thermodynamics, and Isotherms
Source: Molecules. 2023 Oct 17;28(20):7124. doi: 10.3390/molecules28207124 (PMC10608902; doi:10.3390/molecules28207124)
Supplement: Supplementary file 1 [file molecules-28-07124-s001.zip › molecules-2613081-supplementary.pdf]

Ecofriendly Synthesis of Magnetic Composites Loaded on Rice Husks for Acid Blue 25 Decontamination: Adsorption Kinetics, Thermodynamics, and Isotherms

SUPPLIMENTARY DATA

Table S1. Equations used for Calculation of Thermodynamic Parameters

Thermodynamics	(Equation)	Plot	Reference
**Δ G° (KJ/mol)**	Δ G° = -RTln K_L	1/T vs. lnK_L	[1]
**Δ H° (KJ/mol)**	Δ H° = -slope × R		[1]
**Δ S° (J/K*mol)**	Δ S° = intercept × R		

Table S2. Isothermal Models and Equations Applied on Adsorption of Acid Blue 25

Adsorption Isotherms				
	Linear Form	Non-Linear Form	Plot	Reference
**Freundlich**	$\ln(q_e) = lnK_F + \left(\frac{1}{n}\right) \times \ln(C_e)$	$q_e = K_F C_e^{1/n}$	$\ln q_e vs. \ln C_e$	[2]
**Langmuir-I**	$q_e = \frac{q_{mL}.K_L.C_e}{1 + K_L.C_e}$	$\frac{C_e}{q_e} = \frac{1}{q_{mL}} C_e + \frac{1}{q_{mL} K_L}$	$\frac{C_e}{q_e} vs. C_e$	[2]
**Langmuir-II**	-	$\frac{1}{q_e} = \frac{1}{q_{mL} K_L}\left(\frac{1}{C_e}\right) + \frac{1}{q_{mL}}$	$\frac{1}{q_e} vs. \frac{1}{C_e}$	
**Langmuir-III**	-	$q_e = q_{mL} - \left(\frac{1}{K_L}\right)\frac{q_e}{C_e}$	$q_e vs. \frac{q_e}{C_e}$	
**Langmuir-IV**	-	$\frac{q_e}{C_e} = K_L q_{mL} - K_L q_e$	$\frac{q_e}{C_e} vs. q_e$	
**Dubinin–Radushkevich**	$lnq_e = lnq_{mDR} - K_{DR}\varepsilon^2$	$q_e = q_{mDR} e^{-K_{DR}\varepsilon^2}$<br>$\varepsilon = \mathrm{RTln}\left(1 + \frac{1}{C_e}\right)$<br>$E = \frac{1}{\sqrt{2K_{DR}}}$	$lnq_e vs. \varepsilon^2$	[3]
**Temkin**	$q_e = \frac{RT}{B_T} lnK_T + \frac{RT}{B_T} lnC_e$	$q_e = \frac{RT}{B_T} lnK_T C_e$	$q_e vs. lnC_e$	[4]

Table S3. Kinetic Models Applied on Adsorption of Acid Blue 25

**Adsorption Kinetic Models**				
**Pseudo Second Order**		Equation	Plot	Reference
**Linear Forms**	Type 1	$\frac{t}{q_t} = \frac{1}{k_2 {q_e}^2} + \left(\frac{1}{q_e}\right) t$	t/q_t vs. t	[5]
	Type2	$\frac{1}{q_t} = (\frac{1}{k_2 {q_e}^2}) \frac{1}{t} + (\frac{1}{q_e})$	$1/q_t$ vs. $1/t$	
	Type 3	$\frac{q_t}{t} = \mathrm{k}q_e^2 - \mathrm{k}q_e q_t$	q_t/t vs. q_t	
	Type 4	$q_t = q_e - (\frac{1}{\mathrm{k}q_e}) \frac{q_t}{t}$	q_t vs. q_t/t	
**Pseudo Second Order (Non-Linear Form)**		$q_t = \frac{k_2 q_e^2 t}{1 + k_2 q_e t}$	$1/q_t$ vs. $1/t$	[6]
**Pseudo First Order (Linear Form)**		$\ln(q_e - q_t) = \ln(q_e) - k_1 t$	$\ln(q_e - q_t)$ vs. t	[7]
**Pseudo First Order (Non-Linear Form)**		$q_t = q_e(1 - e^{-k_1 t})$	q_t vs. t	[8]
**Intraparticle Diffusion Kinetic Model (Linear and Non-Linear Form)**		$q_t = K_{Diff} t^{1/2} + C$	$q_t vs. t^{1/2}$	[5]
**Elovich Kinetic Model (Linear Form)**		$q_t = \frac{1}{\beta} \ln(\alpha\beta) + \frac{1}{\beta} lnt$	$q_t vs. lnt$	[9]
**Elovich Kinetic Model (Non-Linear Form)**		$q_t = \frac{1}{\beta} \ln(\alpha\beta t + 1)$	$q_t vs. t$	

Table S4. Chemicals and Equipment used for the Work

**Chemical/Equipment**	**Make**
$FeCl_3.6H_2O$	Sigma Aldrich, Germany
NaOH	BDH
Millipore water	Sigma Aldrich, Germany
Acid Blue 25	Sigma Aldrich, Germany
TGA	Schimadzu (model = SDT Q 600 V8.2 Build 100)
FTIR	Model Shimadzu AIM-8800
SEM	JEOL model 2300

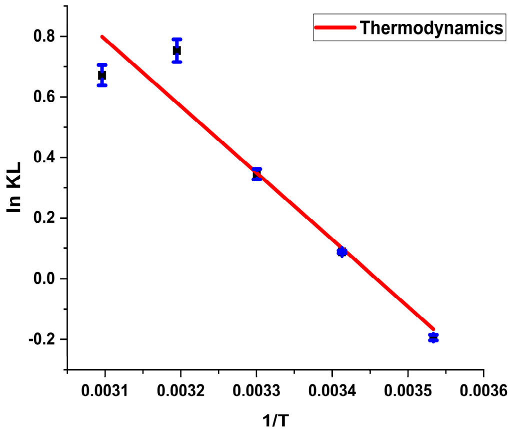

Figure S1. Thermodynamics for Acid Blue 25

REFERENCES

1. Sundaramurthy, S.; Srivastava, V.; Mishra, I., Isotherm, Thermodynamics, Desorption, and Disposal Study for the Adsorption of Catechol and Resorcinol onto Granular Activated Carbon. *J. Chem. Eng. Data.* **2011,** 56, 811-818.
2. Salih, S.; Anwer, S.; Faraj, R., Biosorption of mercury from wastewater using isolated Aspergillus sp. modified 1,10 Phenanthroline: Hill isotherm model. Sci. j. Univ. Zakho . 2017, 288-295.
3. Bayuo, J.; Pelig-Ba, K.; Abukari, M., Isotherm Modeling Of Lead (II) Adsorption From Aqueous Solution Using Groundnut Shell As A Low-Cost Adsorbent. IOSR j. appl. chem. 2018, 11, 18-23.
4. Shafiq, M.; Alazba, P.; Amin, M., Kinetic and Isotherm Studies of Ni2+ and Pb2+ Adsorption from Synthetic Wastewater Using Eucalyptus camdulensis—Derived Biochar. Sustainability 2021, 13, 3785.
5. Oboh, I.; Aluyor, E.; Audu, T., Second-order kinetic model for the adsorption of divalent metal ions on Sida acuta leaves. Int J Phys Sci 2013, 8, 1722-1728.
6. Zafar, S.; Khalid, N.; Daud, M.; Mirza, M., Kinetic Studies of the Adsorption of Thorium Ions onto Rice Husk from Aqueous Media: Linear and Nonlinear Approach. The Nucleus. 2015, 52, 14-19.
7. Abbas, M., Application of Used Tea Leaves for Multi Functions. 2014, II II.

8. Yu, T.; Liang, S.; Shang, X. In Kinetic and thermodynamic study of Am(III) sorption on Na-bentonite: Comparison of linear and non-linear methods. Indian J. Chem. Technol. 2017, 24, 123-133.
9. Benmessaoud, A.; Djamel, N.; Mekatel, E. H.; Samira, A., A Comparative Study of the Linear and Non-Linear Methods for Determination of the Optimum Equilibrium Isotherm for Adsorption of Pb 2+ Ions onto Algerian Treated Clay. Iran. J. Chem. Chem. Eng. 2020, 39, 153.
